# A case of anal melanoma treated with wide local excision and doublet immunotherapy

**DOI:** 10.1093/jscr/rjag375

**Published:** 2026-05-16

**Authors:** Mohammad Nizamuddin, Lina Issa, Angeline Rivkin, Sebastian Valdivieso, Aslam Ejaz, Mohamad Abdulhai

**Affiliations:** College of Medicine, University of Illinois, 1853 W Polk St, Chicago, IL 60612, United States; College of Medicine, University of Illinois, 1853 W Polk St, Chicago, IL 60612, United States; Division of Colon and Rectal Surgery, Department of Surgery, University of Illinois, 1801 W. Taylor St. Chicago, IL 60612, United States; Division of Colon and Rectal Surgery, Department of Surgery, University of Illinois, 1801 W. Taylor St. Chicago, IL 60612, United States; Division of Surgical Oncology, Department of Surgery, University of Illinois, 1801 W. Taylor St.Chicago, IL 60612, United States; Division of Colon and Rectal Surgery, Department of Surgery, University of Illinois, 1801 W. Taylor St. Chicago, IL 60612, United States

**Keywords:** anal melanoma, immunotherapy, multidisciplinary approach

## Abstract

Anal melanoma is one of the rarest forms of anal cancer and melanoma. Due to its rarity and non-specific symptoms, many cases undergo a prolonged course before diagnosis. In our case study, we present a 55-year-old female diagnosed with anal melanoma with bilateral inguinal lymph node involvement. Doublet immunotherapy followed by wide local excision was successful in treating the disease without signs of recurrence to date.

## Introduction

Anal melanoma is a rare and aggressive malignancy accounting for 1%–2% of anorectal cancers, and less than 1% of melanomas [1]. Due to its rarity and generally nonspecific presentation, diagnosis often occurs at an advanced stage, with more morbid outcomes. It typically presents in older adults, with a higher rate in women [2].

Anal melanoma involves the malignant transformation of melanocytes in the anal region. It is typically characterized by atypical melanocytes, with immunohistochemical positivity for S100, HMB-45, and Melan-A biochemical markers [3]. The prognosis for patients with anal melanoma is poor, with 5-year survival rates ranging between 10% and 20% [4]. Surgical treatment is the definitive treatment with lymph node dissection and adjunct immuno or radiotherapy [5].

Surgical treatment for anal melanoma historically involved abdominoperineal resection (APR). However, the recognition in cutaneous melanoma that survival is driven by systemic disease rather than local control has led to the de-escalation of surgery. Historically, wide local excision and APR were the standard surgical options, with no significant difference in overall survival noted, although some reviews have shown lower recurrence rates noted in APR, which must be managed with higher morbidity and lower quality of life [6]. Studies support this notion, as it has been shown that APR holds no overall survival benefit as compared to local excision for anal melanoma [7].

Due to its aggressive nature, with poor outcomes and rarity, we highlight the case of a 55-year-old female with anal melanoma and bilateral lymph node involvement who was treated with curative surgery and immunotherapy.

## Case report

**Figure 1:**
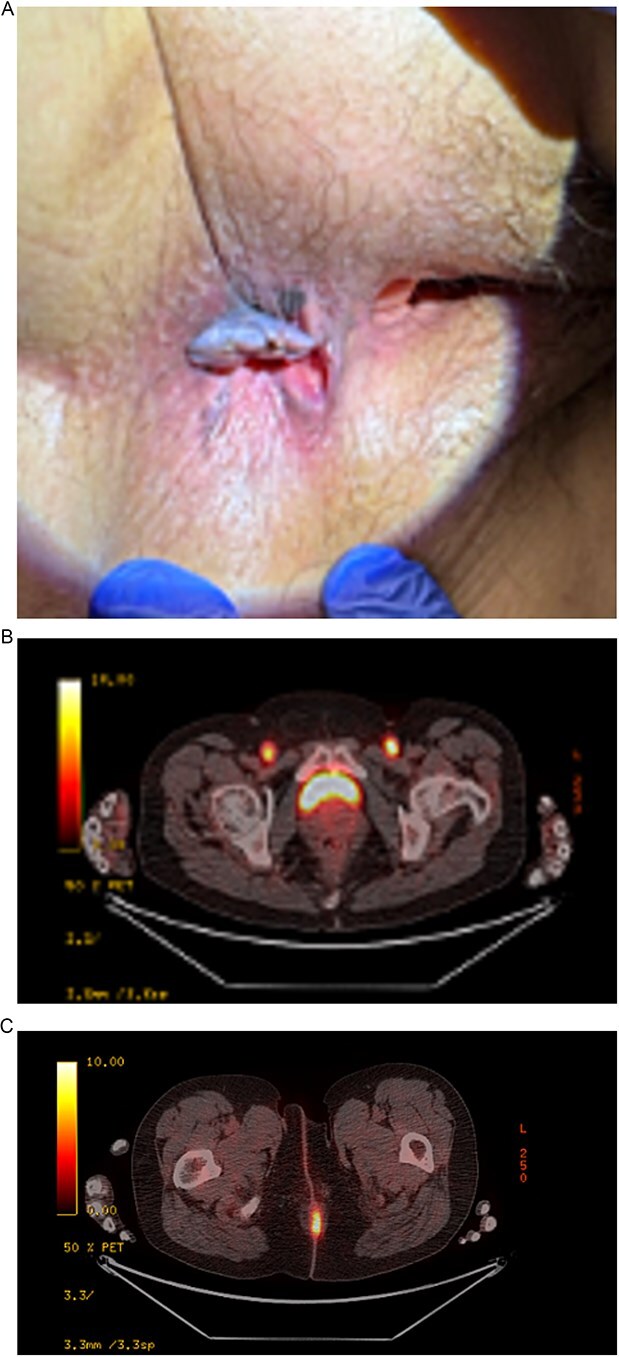
(A) 1 × 1 cm melanocytic mass in the right posterior peri-anal aspect. (B) PET/CT scan displaying bilateral FDG-avid inguinal lymphadenopathy with increased uptake. (C) Uptake in anal primary lesion.

A 55-year-old female patient presented to the clinic for right inguinal swelling, in which a CT scan at an outside hospital performed showed lymphadenopathy. Subsequent IR biopsy to evaluate her right inguinal lymphadenopathy confirmed malignant metastatic melanoma. A PET-CT scan showed bilateral FDG-avid inguinal lymphadenopathy with a focal area of thickening and increased uptake at the anal/perianal regions, but no distant metastasis. She had no significant past medical history, along with no family history of colorectal cancer, no prior abdominal surgeries, and no prior colonoscopy. In retrospect, she informed providers that she had noticed a perianal lump with painful bleeding that she attributed to hemorrhoids. On physical exam there was a large palpable right inguinal lymphadenopathy and a smaller palpable left inguinal lymph node. A perianal mass in the right anterior aspect was visualized with melanotic coloration, appearing ulcerated. DRE revealed some firmness in the posterior midline (Fig. 1).

**Figure 2:**
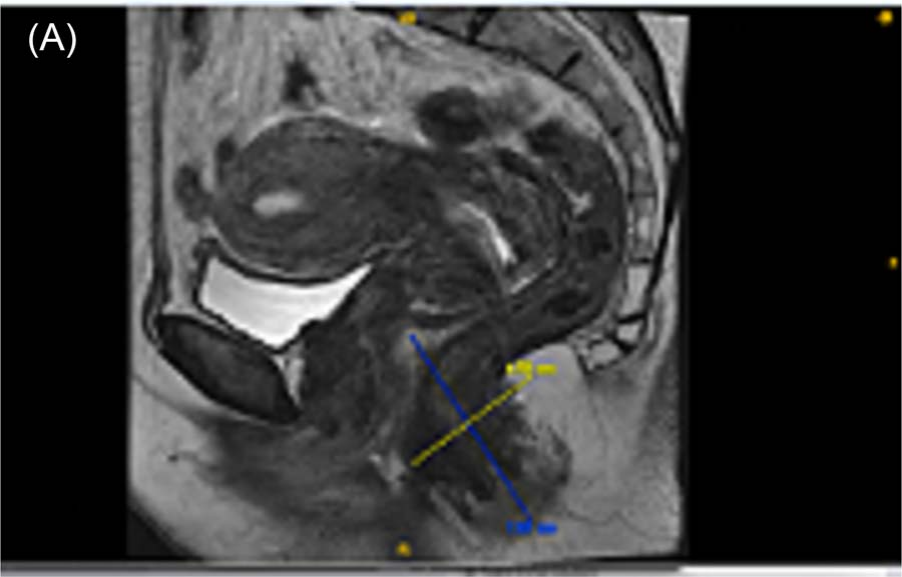
MR pelvis with and w/o contrast [MN2]. (A) MRI demonstrating T1 Hyperintense anal lesion with restricted diffusion and early contract enhancement.

The patient was discussed at a multidisciplinary tumor board and started neoadjuvant immunotherapy for stage II anal melanoma. Due to lack of guidelines for anal melanoma we extrapolated based on existing NCCN cutaneous melanoma guidelines, she was given two cycles of doublet immunotherapy with pembrolizumab and nivolumab with subsequent restaging. The patient had symptomatic improvement with resolution of pain and bleeding, as well as adequate clinical response on physical exam. Follow-up MRI of the pelvis showed a residual 1.6 cm lesion at the anal verge, with restricted diffusion and bilaterally enlarged inguinal lymph nodes (Fig. 2).

**Figure 3:**
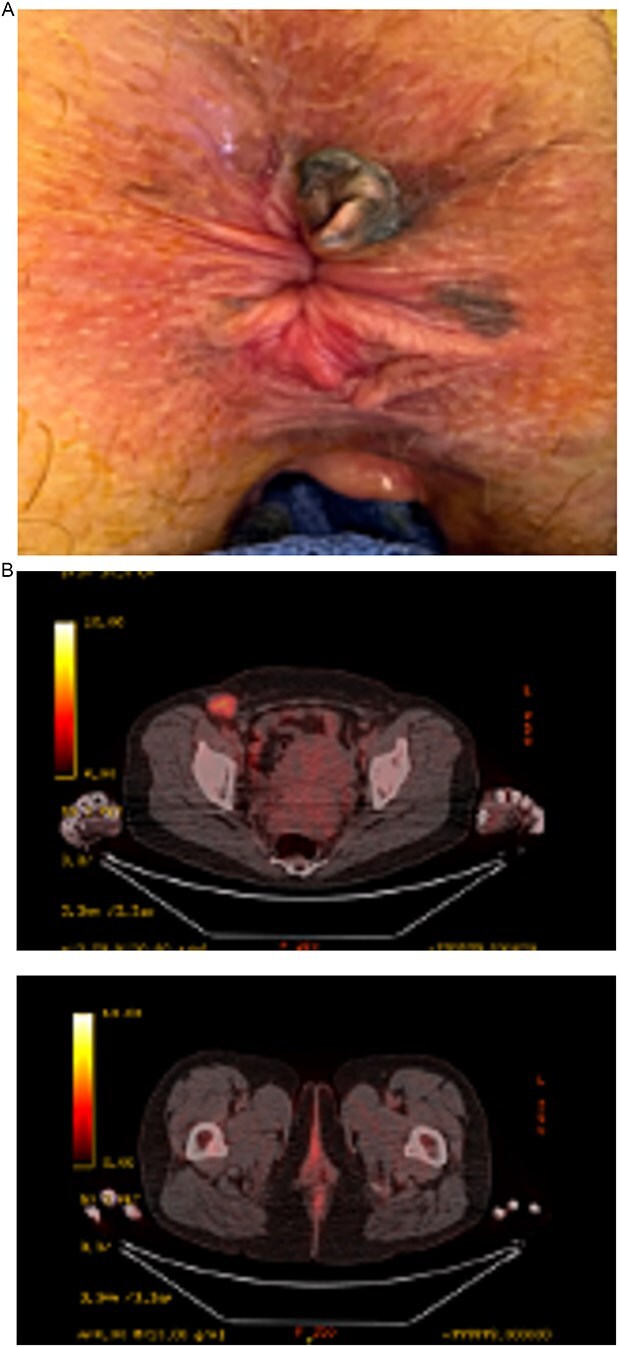
(A) Post-immunotherapy changes of lesion. (B) Post-immunotherapy PET scan showing decrease in metabolic activity with interval progression bilaterally of bilateral inguinal lymphadenopathy.

Repeat PET-CT imaging showed reduced metabolic activity in the anal lesion and no distant disease. An examination under anesthesia with endoanal ultrasound indicated clinical response to the immunotherapy regimen. EUS identified a 1 × 1 cm melanotic mass at the right posterior perianal aspect over the intersphincteric groove, a 1 cm × 5 mm melanotic lesion in the lateral location, and a 4 × 3 mm lesion at the dentate line. EUS showed possible involvement of the internal sphincter muscle fibers by the posterior lesion, but no evidence of external sphincter involvement (Fig. 3).

**Figure 4:**
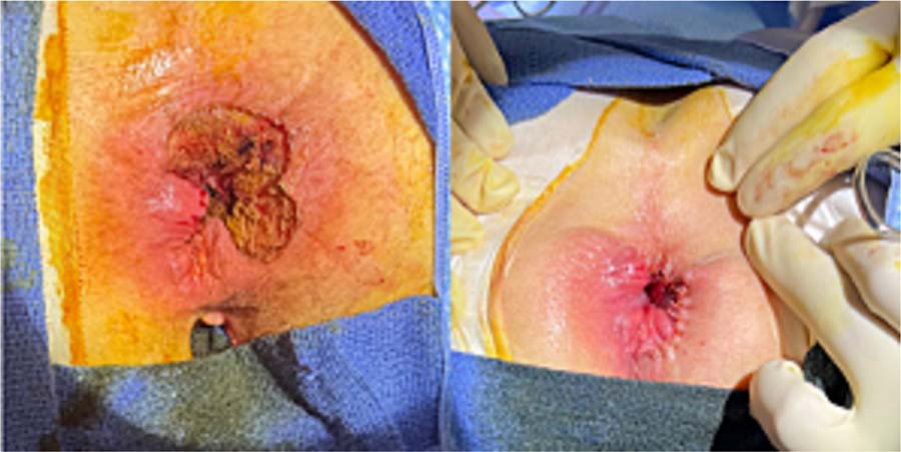
Shows status post wide local excision of all lesions with primary closure.

The patient underwent successful wide local excision of all identified anal lesions with preservation of the external sphincter muscle and bilaterally targeted inguinal lymph node dissection. This patient had an uncomplicated postoperative recovery. Outpatient follow-up visits indicated healed surgical wounds with no complications. Final pathology labeled the disease as ypT0N1b and showed no residual melanoma in the primary lesion, with a 2.2 mm deposit in one of the right inguinal lymph nodes positive for SOX10, from the 9 lymph nodes dissected. The patient was discussed again at the tumor board, and the decision was made to proceed with adjuvant immunotherapy for 1 year along with surveillance (Fig. 4).

## Discussion

Anal melanoma is a rare and aggressive malignancy that presents with nonspecific symptoms, such as rectal bleeding and perianal masses, which can often go undetected, leading to difficulty in treatment, which is compounded by lack of anal melanoma specific guidelines. The management of anal melanoma has been largely influenced by the management of cutaneous melanoma. Cutaneous melanoma was the first solid tumor where modern immunotherapies such as checkpoint inhibitors (e.g. anti-PD-1 and anti-CTLA-4) showed survival benefits [8]. With the advent of immunotherapy, surgical management has shifted to a more conservative approach, utilizing wide local excision rather than APR, which allows for sphincter preservation and improved quality of life, due to studies showing distant metastasis in patients even with APR [9].

Our patient underwent immunotherapy, surgical excision, and lymph node dissection. Our approach was guided by evolving guidelines, including the institution of wide local excision for anal melanomas. By utilizing doublet immunotherapy for neoadjuvant therapy, we demonstrated significant clinical efficacy with notable reductions in the metabolic activity and size of the primary lesion.

Inguinal lymph node involvement indicated metastatic involvement of the anal melanoma, necessitating regional lymph node management early in the disease. This was done via bilateral lymphadenectomy, as the pathology reports indicated minimal metastatic deposits in lymph nodes and no residual melanoma. Given the high recurrence rate of anal melanoma, this patient will be followed closely, with her most recent post-op visits indicating no recurrence of disease.

## Conclusion

This case underscores the evolving therapeutic landscape of anal melanoma and highlights how treatment paradigms derived from cutaneous melanoma may help in the setting of limited disease-specific guidance. This case highlights the importance of early suspicion and biopsy for abnormal perianal lesions, multidisciplinary management teams to diagnose and treat the condition with the advent of immunotherapy in anal melanoma, and close post-operative follow-up and surveillance.

## Data Availability

The data supporting this study’s findings are available from the corresponding author upon request.
